# A randomized effectiveness trial of individual child social skills training: six-month follow-up

**DOI:** 10.1186/s13034-014-0031-6

**Published:** 2014-12-23

**Authors:** John Kjøbli, Terje Ogden

**Affiliations:** The Norwegian Center for Child Behavioral Development, University of Oslo, P.O. Box 7053, Majorstuen, 0306 Oslo Norway

**Keywords:** Conduct problems, Child social skills training, Effectiveness study, Randomized controlled trial

## Abstract

**Background:**

Individual Social Skills Training (ISST) is a short term, individually delivered intervention (8-10 sessions) that promotes social skills in children with emerging or existing conduct problems. This study examined the effectiveness of ISST immediately and 6 months after the termination of the intervention.

**Methods:**

The participants were 198 children (3-12 years) who were randomly assigned to ISST or practice as usual. The data were collected from parents, children and teachers.

**Results:**

Findings showed positive change on most outcomes in both study conditions. However, examining the relative effectiveness of the intervention, only one positive effect of ISST emerged on parent-reported child conduct problems immediately after intervention.

**Conclusions:**

These results suggest that compared to the control group, ISST had limited effects in ameliorating child problem behavior. These data suggest that it is not sufficient to provide ISST when aiming to reduce conduct problems in children.

**Electronic supplementary material:**

The online version of this article (doi:10.1186/s13034-014-0031-6) contains supplementary material, which is available to authorized users.

## Background

Longitudinal studies have shown that adolescent and adult antisocial behavior and criminal involvement often have roots in conduct problems that begin in early childhood. Both individual risk factors (e.g., undercontrolled temperament, attention problems and delayed motor development) and social factors (e.g. parental neglect of child, inconsistent and harsh discipline) are important to the emergence and persistence of conduct problems [1]. Fortunately, extensive research has shown that evidence-based parent training interventions are effective in reducing child conduct problems [2,3]. However, the effects of parent training do not necessarily diffuse to settings other than the home [4,5]. Interventions that directly target children are important when parents can or will not participate in parent training due to contextual factors, such as life stress, work conflicts, family issues, interpersonal issues and parental psychopathology [6,7]. Furthermore, children with conduct problems are at risk of being rejected and disliked by peer students as early as preschool and have been found to actively seek peers who are behaviorally similar to themselves [8]. These findings have inspired the development of Individual Social Skills Training (ISST), which is one of the interventions in the multi-modular program Early Initiatives for Children at Risk (Norwegian acronym, TIBIR). This program was developed with the aim of scaling up the use of evidence-based interventions for the prevention and reduction of conduct problems in children [9]. In addition to ISST, TIBIR also consists of three parent training interventions and one teacher training intervention [9]. These interventions are tailored based on the severity of the children’s conduct problems. ISST is always offered in combination with parent training, as TIBIR builds on the principles of the social interaction learning (SIL) model, suggesting that conduct problems are caused by coercive and aggressive parent-child interactions [10]. Another overriding principle of TIBIR is that all interventions should be evidence-based; currently three have been evaluated in effectiveness trials [4,11,12]. Therefore, in an effort to evaluate the effects of every intervention in TIBIR, the present study tested the unique effectiveness of ISST (without parent or teacher training) in a randomized, controlled trial immediately following and six months after the completion of the intervention. The fact that ISST was tested in an effectiveness trial (i.e., real world settings), and not in an efficacy trial (i.e., optimal settings), makes it likely that the findings from the current sample are generalizable to the population of Norwegian children with conduct problems.

### Individual social skills training

Social skills training is often conducted as a group intervention in which children are taught social and cognitive skills (i.e., play, friendship and conversational skills, problem solving, self-control, anger management, empathy training, and perspective taking). However, social skills training may also be individually delivered to children to avoid negative group influences. Group-based social skills training in homogeneous groups of children and youth with conduct problems may produce unintended negative outcomes; this result is often referred to as deviance training [13,14].

Individual training by *coaching* is designed to teach children the skills they need for social acceptance and friendship through techniques such as discussion, rehearsal, and feedback from the coach. The advantage of individual training is that skills may be tailored and selected after considering what skills would be most important for the young person to learn. Based on the theories underlying social skills training, it has been assumed that learning individual social skills should result in a reduction of externalizing problem behavior. However, the empirical support for this assumption has turned out to be rather weak [15,16].

### Research on social skills training

In general, the findings regarding child social skills training interventions have been mixed. Some have been positive, such as the findings that showed that children who received The Incredible Years child training had significantly lower parent-reported externalizing problems and less teacher-reported aggression immediately after treatment compared to controls. At follow-up one year later, most of treatment effects had been maintained [6]. In a more recent study, the same authors found that child training had a significant effect on children’s social competence that generalized from the school to the home setting [7]. Similarly, Kazdin, Siegel and Bass conducted an RCT that evaluated Problem-Solving Skills Training and a parent-management training intervention and found the outcomes to be similar across settings (home, school and in the community) at post-treatment [17]. One year later, the effects of the child training had been maintained compared to parent management training to a large degree.

Meta-analyses have indicated that social problem-solving training on its own primarily strengthens social-cognitive skills, compared to social skills training, which primarily improves social interaction skills [18,19]; neither form of training influenced child problem behavior significantly. Schneider concluded in his review of 79 social skills programs that they generally had positive effects, but the outcomes were more positive when offered to withdrawn children than to unpopular or aggressive children [20]. Younger children, immature children and aggressive children seemed to profit least from social skills training because either the contents or the presentation were insufficient or inadequate [21]. In a systematic review, social competence training seemed to be most effective for children who had been exposed to critical life events and who lacked social stimulation, while children with externalizing and internalizing problems had approximately the same moderate outcomes [19]. Children who were anxious, isolated and lonely seemed to benefit more than those conduct problems. A later meta-analysis demonstrated that social competence training had a small short-term effect on a broad range of behavioral and mental health problems and the long-term outcomes were smaller [22]. In this review, the average post-test effect size (Cohen’s d) was .29 and the follow-up effect size was .21. These cognitive-behavioral programs turned out to produce the most sustainable outcomes for children with conduct problems, but the effect sizes were higher for social competence outcomes than for antisocial behavior outcomes. This review demonstrated that few studies assessed follow-up outcomes. Furthermore, the findings showed that most studies included small samples (70.8% had fewer than 49 participants), and only a few of the studies (10.4%) evaluated individually delivered social skills training. Kavale, Mathur, Forness, Rutherford & Quinn conducted a meta-analysis of 64 single-case studies of social skills training of children with social and emotional difficulties [23]. The discouraging conclusion of this study was that individual social skills training had little support in research, even when it was frequently applied to this target group. In another meta-analysis, Quinn, Kavale, Mathur, Rutherford & Forness found a positive but small effect of individually delivered social skills training but concluded that such training on its own was not sufficient to prevent or reduce child conduct problems [24]. An increase in social competence did not automatically result in reduction of conduct problems.

According to the literature, the greatest challenges to social competence training seem to be the lack of environmental support, which limits the generalization or transfer of training effects and lowers the sustainability of the program activity and outcomes [21]. Formal monitoring of program implementation and control of intervention integrity is also often missing. It is often difficult to confirm whether a program has been delivered with the dosage and engagement that was intended by the program developer. The social validity (i.e., the degree to which an intervention is socially acceptable and/or relevant) of some programs has also been questioned, indicating that they apply to few of the skills that make up social competence.

In sum, social skills training has produced varying results in outcome studies, and children with conduct problems generally seem to benefit less than children with internalizing problems. The modest outcomes may be related to the limited content or duration of training, the mismatch of the intervention to the children’s needs, and the weakness of the evaluation designs (e.g., short time frame, lack of follow up studies, and few evaluations of the implementation quality).

Although the findings have been mixed [25,26], there are indications of increased effects of social skills training when combined with behavioral parent training [7,17,27]. When the promotion of cognitive and social competences is integrated with changing family interactions and increasing the quality of parenting, long-term protective effects have been demonstrated [28]. While such studies are important, it is still necessary to examine whether social skills training has unique effects because previous evaluations have revealed mixed results, and it remains an open question whether social skills training adequately impacts conduct problems. To examine the unique effect of individually delivered social skills training on conduct problems, the current study evaluated the effectiveness of Individual Social Skills Training (ISST). Previous trials have largely been conducted within small samples efficacy trial designs and few trails have included a practice as usual comparison group. Thus, the current randomized effectiveness trial adds to the existing literature by including a relatively large sample, follow-up assessments, a practice as usual comparison group and measurement of intervention integrity.

### Aims

The aim of the present study was to evaluate the effectiveness of ISST, compared to practice as usual, immediately and six months after intervention in real-world settings in a sample (N = 198) of children with emerging, or already developed, conduct problems. More specifically, the main aim of the present study was to examine whether the intervention would have immediate and long-term positive effects on child conduct problems and social competence. The second aim was to examine whether any effects would emerge on co-occurring internalizing problems in children with conduct problems [21].

## Methods

The design of the study was a pre-test, post-test and follow-up control-group randomized trial with a 50:50 allocation ratio between the intervention and the comparison groups. The children were the units of analyses.

### Participants

**Figure 1:**
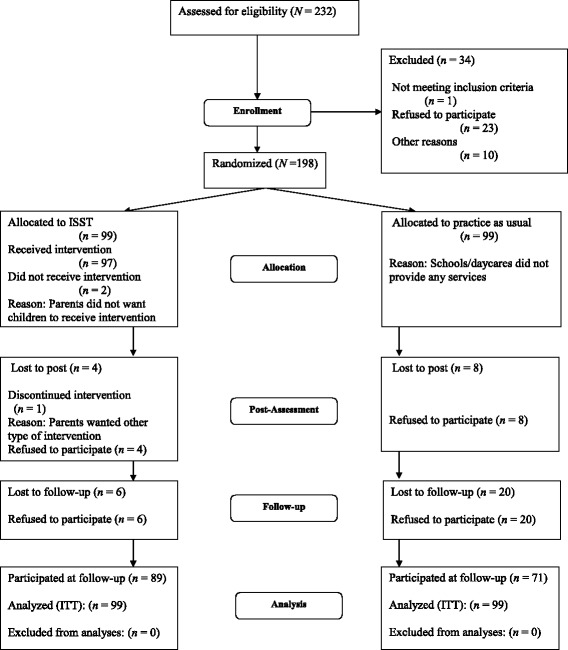
**Flowchart of the ISST effectiveness study.** ITT = Intent-to-treat.

The calculations for statistical power indicated that approximately 200 children would be needed to identify significant differences between the ISST and the comparison group. The sample size was based on the minimum sample required to be reasonably likely to detect a small to moderate effect (Cohen's d = .40). This effect size was derived from the abovementioned meta-analysis, indicating that the average effect size ranged between .29 to .21, [22] and two evaluations of SNAP where effect sized ranged between .79 to 1.2 [29,30]. Although the meta-analysis indicated that a larger sample size was needed, both the SNAP studies and practical reasons (i.e., time and resources) made us set the effect size at .40. Type 1 error probability α = .05, Type 2 error probability β = .20, power (1 - β) = .80 (two-tailed test). Thus, 232 children were assessed for eligibility (see Figure 1). Hundred and ninety-eight families agreed to participate in the pre-test assessment. The families were recruited from 9 municipalities in Norway. The participating children (3-12 years) were either at an early stage of development of problem behavior or developing conduct problems. To match the regular procedures for referral or intake to municipal and schoolchild services, no formal screening procedures were used as part of this study. Thus, the intervention was offered based on practitioners’ judgments.

Children between the ages of 3 and 12 who exhibited problem behaviors (e.g., aggression, delinquency, or externalizing behavior) at daycare or school were eligible to participate in the present study. Children were excluded from participation if they (a) were diagnosed with autism, (b) had been exposed to documented sexual assaults, (c) were mentally retarded, or (d) had parents with serious mental health problems or severe intellectual disabilities (see Figure 1). One child was excluded because of these criteria (see Figure 1).

The 198 children in this study ranged from 3 to 12 years of age at intake (*M* = 7.64, *SD* = 2.19), and 39 (19.7%) were girls. The average age of the reporting parent was 36.30 years (*SD* = 6.07). Most of the parents reported to have Norwegian background (182 or 92%), but two (.1%) were from another western European country, and 14 (7.1%) reported “other” ethnicity. The average gross annual family income was 564,088 Norwegian Kroner (*SD* = 267.049), which is approximately $96,756. Among the participating children, 106 (53.5%) lived with both biological parents, 29 (14.6%) lived with parents who were married or cohabiting with another adult and 63 (31.8%) lived with single parents (divorced, separated, or never married). The proportion of single parents was markedly higher than that of the general population (20.1%) [31]. According to the parents’ self-reports, 80 (40%) had a college or higher university degree, 96 (48.5%) had finished high school, and 22 (11.1%) had completed junior high school or elementary school.

### Procedures

The project was approved by the regional ethics board. The families were assessed from September, 2007 to June, 2008. The eligible families were informed of the study and agreed to participate by signing a written informed consent document. The participating families were assessed before (pre-test), immediately after (post-test), and 6 months after (follow-up) the completion of the intervention. The assessment sessions, that took place at the agencies where the interventions were offered, were administered by research staff employed and trained for the purposes of collecting data for this study. These data assessors, who were social workers, did not administer any of the interventions offered in this study nor did they hold any particular preference for any given intervention. They did not have access to the data, as these were filled out on a computer program only accessible to the informants. The informants were informed that the research staff could not access the data. The parents completed questionnaires about family demographics and child behavior (on laptops). If the parents agreed (all provided consent), each child’s teachers were informed of the study and asked to complete questionnaires about each child's behavior. Every child aged seven years or older was informed of the study and asked to complete questionnaires about their social competence and internalizing problems. The family assessment sessions lasted for approximately one hour. For budgetary and logistic reasons, only the parent who reported having spent the most time with the child was asked to fill out the questionnaires. The parents were not offered economic compensation for participating in the study.

After completing the pre-test, the families were randomly assigned to the intervention condition or the comparison condition (i.e., practice as usual; see Figure 1). The random assignment procedure was computer-generated by a staff member who did not recruit the families and who was monitored by the principal investigator of the study. When the randomization of the families had been carried out, the local interventionists were informed about which families should receive ISST and which should receive regular practice. No interim analyses were conducted during the trial. The recruitment of children ended when the participating agencies had reached 198 families.

### Intervention

The aim of ISST is to teach at-risk children in preschools and schools to increase their social skills and reduce their conduct problems by lowering the reinforcement of antisocial strategies and contact with deviant peers and by reinforcing the use of pro-social skills. ISST is a cognitive-behavioral intervention that is flexible and individually tailored to match the age appropriate behaviors and needs of the children. Thus, the intervention dosage varies from one case to another. Although ISST is individually tailored, all children receiving the intervention are taught to stop, think and develop socially appropriate plans before they act, in order to cope with anger and to reduce aggressive and negative behaviors. Throughout the intervention, children are encouraged to learn and role-play emotion regulation, problem solving skills and anger management skills. A key aspect of the intervention is to help children identify cues and triggers in the environment that make them angry or distressed, and to teach them to be aware of bodily sensations when faced with such triggers, so that they can calm down in order to make a plan before engaging in a behavior that can have negative outcomes for the child and others. For instance, if a child frequently gets into fights at the playground, the social skills trainer will teach ways to behave appropriately in this context. This will be achieved by helping the child understand when and where the problems begin, to problem solve how to avoid problems and to role-play good ways of behaving at the playground. The principles for this intervention are derived from the SIL-model [9] and Stop Now and Plan (SNAP™), a cognitive behavioral strategy developed by the Child Development Institute [32]. ISST is a short-term intervention, intended to last for approximately 8-10 weeks, with one session per week (M = 8.59 hours), delivered in the school or preschool setting.

The social skills trainers were assistants and other personnel such as social workers working directly with children in schools and daycares. A manual was developed to secure adherence (e.g., by illustrating how to teach children social skills by the use of role-play and encouragement) [33]. The social skills trainers were trained and coached individually for six days spread over six months and received case supervision by a trained therapist. The training of the social skills trainers was standardized and consisted of:

- Lectures
- Role-play exercises
- Home assignments about child conduct problems and their causes, manifestations and developmental trajectories
- Training in social skills relevant for children in the target group (e.g., anger management, problem solving and cooperation).

In all, 64 social skills trainers participated in the present study (36 had one child, 25 had two and three had three).

The participants in the comparison group were free to seek and receive any available intervention offered in school and/or regular services. The parents reported that 18.6% of the children in the comparison group received special education commonly offered in the school context in Norway (received special education, mainly in regular subjects like Norwegian or mathematics, or attended a special class) during the study period. We did not collect detailed information about the content of the interventions provided in the comparison group.

### Measures

The parent-reported child outcomes were measured with the Eyberg Child Behavior Inventory (ECBI) [34], the Home and Community Social Behavior Scales (HCSBS) [35], and the Child Behavior Check List (CBCL) [36]. The ECBI is widely used and consists of 36 items (e.g., “Has temper tantrums”) that have been translated to Norwegian and standardized with a Norwegian child sample [37]. The instrument consists of an intensity scale that indicates the frequency of conduct problems (7-point Likert-scale items) and a problem scale that indicates whether the reporting parent experiences the behaviors of the child as problematic (scored as 1) or not problematic (scored as 0). The Cronbach’s alphas for the intensity scale pre-test, post-test and at follow-up were .93, .92 and .93, respectively. The Cronbach’s alphas for the problem scale were .92, .92 and .92, respectively. The HCSBS inventory consists of 64 items (rated on a 5-point Likert scale) that indicate both conduct problems (32 items, e.g., “Gets into fights”) and social competence (32 items, e.g., “Is accepting of peers”). The Cronbach’s alphas for the conduct problems pre-test, post-test and at follow-up were .94, .94 and .95, respectively, and the Cronbach’s alphas for social competence pre-test, post-test and at follow-up were .94, .95 and .95, respectively. The HCSBS was translated into Norwegian at the Norwegian Center for Child Behavioral Development [38]. The CBCL was used to measure child anxiety and depression. The CBCL has been validated and standardized in a Norwegian study [39]. The Anxious/Depressed Scale (14 items; Cronbach’s alphas were .84, .84 and .83, respectively; item example, “Worries”) consists of 3-point Likert-scale items to which the respondent can answer “0” if the item is never/seldom true of the child, “1” if the item is sometimes or somewhat true, and “2” if the item is often or always true.

The teacher-reported child outcomes were measured with the School Social Behavior Scales (SSBS) [35] and the Teacher Report Form [36]. The SSBS inventory was translated to Norwegian at the Norwegian Center for Child Behavioral Development and consists of 64 items (5-point Likert scale) that indicate both conduct problems (32 items, e.g., “Insults peers”) and social competence (32 items, e.g., “Cooperates with other students”). The Cronbach’s alphas for the conduct problems pre-test, post-test and follow-up were .96, .97 and .96, respectively, and Cronbach’s alphas for the social competence were .94, .96 and .97, respectively. The TRF was used to measure anxiety and depression. The TRF has been validated and standardized in a Norwegian study [40]. The Anxious/Depressed Scale (18 items; Cronbach’s alphas were .85, .84 and .85, respectively; item example, “Afraid of mistakes”) consists of 3-point Likert-scale items.

The child-reported outcomes were measured with the Loneliness in Children Questionnaire [41], which is a 24-item measure that assesses children's internalizing problems of feelings of loneliness and social dissatisfaction. The Cronbach’s alphas for this measure pre-test, post-test and at follow-up were .89, .89 and .91, respectively. Child social competence was assessed with the Social Skills Rating System (SRSS), which is a 34-item measure that has been revised and translated by Ogden [42]. The SRSS had Cronbach’s alphas pre-test, post-test and follow-up of .89, .91 and .92, respectively. As noted above, only children aged seven years or older were asked to complete these questionnaires.

Adherence to ISST was self-completed by the ISST interventionists at post-test with an instrument developed at the Norwegian Center for Child Behavioral Development specifically for this study. No independent checks were included to validate the adherence measure. The measure consisted of 35 items (scored on a 5-point Likert scale) that indicated the degree to which the ISST interventionists covered the topics and the core components of the intervention (e.g., “I have used reward systems when practicing new skills”, and “We have practiced (role-played) how the child should relate to diverse situations”). The Cronbach’s alpha was .83.

### Analytic procedures

The analyses were performed in PASW (formerly SPSS), version 18. Student’s t-tests (independent sample) and chi-square tests were performed to examine the differences between the intervention and comparison groups at baseline. Linear mixed models (LMMs) were used in intent-to-treat (ITT) analyses to examine intervention effects (from pre-test to post-test and from pre-test to follow-up). The ITTs included all cases of participation at post-assessment or follow-up assessment. The LMMs were run with the entire sample, and the cases with completely missing data were analyzed with estimated values/scores. The LMMs have the advantage of using all available data to account for the correlation between repeated measurements on the same subject, to model time effects, and to handle the missing data more appropriately than traditional ANOVAs [43]. In contrast to traditional ANOVAs where only complete cases are included or the last observation carried forward procedure is applied, LMM takes all available observations (i.e., the direct likelihood method) into account in the analyses. The magnitude of the outcomes was estimated by calculating effect sizes (Cohen’s *d*).

### Missing data, normality, and outliers

Outliers were examined at all assessments to ensure that these values were within the range of scores defined by the maximum and minimum values of the scales. The 5% trimmed mean was compared to the original mean, and in all cases, the differences were small, indicating that the outliers had little impact on the original mean. Therefore, the outliers were not modified.

All scales were examined with regard to a normal distribution and were found to be within an acceptable range of skewness and kurtosis (+/−2). Consequently, no transformations of variables were conducted. To examine multivariate normality, the Mahalanobis distances were calculated for the dependent variables; this test showed that the data contained few outliers, suggesting that no transformations of the data were needed [44].

## Results

### Attrition

Of the 198 participants at pre-assessment, 186 (93.9%) participated at post-assessment, while 160 (81%) participated at follow-up. There was a significant differential attrition rate for the participants in the two groups, *χ*^2^ (1, N = 198) = 10.55, *p* = .01. Of the participants who completed all assessments, 89 (55.6%) were randomized to ISST and 71 (44.4%) to the comparison group. The comparisons at Time 1 (t-tests and chi square tests) between the attrition group and those who completed all assessments showed no differences at intake on the demographic characteristics (child age, child gender, ethnicity, number of siblings, single-parent household, education, family income, or parent age) and the outcome variables, except that the parents who completed all assessments reported significantly lower pre-scores on ECBI intensity, *t*(198) = 2.16, *p* = .03.

### Baseline comparisons

To test for differences between the intervention conditions at pre-assessment, the participants who completed all of the assessments were compared on demographic characteristics and outcome variables. No significant differences emerged among the demographic variables or the parent-, child or teacher-reported outcomes.

### Dosage and fidelity

Dosage in the ISST group was measured by the social skills trainers’ reports of the registered number of sessions they had completed with each family. On average, children in the ISST group received 8.59 hours (*SD* = 1.77). The practitioner adherence to the ISST was found to be high, with a mean score of 4.39 (*SD* = .34) out of a maximum score of 5. The dosage and type of intervention in the comparison group was not collected because of budgetary and logistical reasons. As noted, children in this group could receive any intervention offered as part of practice as usual in school or other mental health services during the study.

### Intervention effects

To test for intervention effects, LMMs were run using the ITT approach. On all variables included in this study, except TRF Anxiety/Depression (p = .27) and SRSS social competence (p = .09), the results revealed significant general time effects. Generally, the findings showed a decrease in conduct problems and internalizing problems, and an increase in social competence across the ISST and the comparison group from pre-test to follow-up. As shown in Table 1, zero of ten Intervention x Time effects were significant, indicating that the intervention and comparison group did not have different slopes from pre-test to follow-up on these outcomes. An examination of the intervention effects at post-test revealed that parents in the ISST group reported that the children presented fewer problems on ECBI intensity, whereas no other measure showed any significant differences between the groups at post-test or at follow-up.

**Table 1:** **Estimated means, standard errors, F-values, t-values and p-values**

	**Intervention group**			**Comparison group**			**Omnibus** **test**		**Post-** **test**		**Follow-up**	
**Variables**	**T1** ***Mean (SE)***	**T2** ***Mean (SE)***	**T3** ***Mean (SE)***	**T1** ***Mean (SE)***	**T2** ***Mean (SE)***	**T3** ***Mean (SE*** **)**	***F***	***p***	***t***	***p***	***t***	***p***
*Parent reported outcomes*												
ECBI Intensity	120.72 (2.99)	106.14 (2.55)	102.20 (2.79)	122.38 (2.99)	113.51 (2.58)	106.83 (2.91)	2.35	.10	2.14	.03	.98	.33
ECBI Problem	12.58 (.87)	8.76 (.80)	7.81 (.76)	12.35 (.87)	9.48 (.81)	7.93 (.79)	.63	.54	1.04	.30	.35	.73
Merrell externalizing	75.17 (2.09)	68.61 (1.89)	66.14 (1.99)	76.63 (2.09)	70.21 (1.91)	68.22 (2.09)	.04	.96	.08	.93	.28	.78
Merrell social competence	105.08 (1.97)	109.42 (2.05)	113.36 (2.08)	104.68 (1.97)	110.21 (2.08)	113.13 (2.20)	.16	.86	.52	.60	.07	.94
CBCL Anxiety/Depression	5.33 (.47)	4.69 (.45)	4.47 (.43)	5.13 (.47)	5.07 (.46)	4.61 (.46)	.84	.44	1.29	.19	.63	.52
*Teacher reported outcomes*												
Merrell externalizing (Teacher)	89.85 (2.61)	85.39 (2.91)	77.07 (2.73)	87.59 (2.59)	84.89 (2.95)	74.43 (2.88)	.24	.79	.59	.56	.10	.92
Merrell social competence (Teacher)	86.44 (2.02)	93.36 (2.36)	95.99 (2.69)	90.52 (2.01)	94.61 (2.39)	97.63 (2.84)	.75	.48	1.19	.24	.70	.48
TRF Anxiety/Depression	8.30 (.57)	7.68 (.58)	7.58 (.60)	7.63 (.57)	7.20 (.59)	6.93 (.64)	.04	.96	.25	.80	.03	.97
*Child reported outcomes*												
SSRS Social competence	99.50 (1.94)	101.35 (2.08)	101.02 (2.22)	93.92 (1.91)	97.60 (2.00)	96.78 (2.22)	.27	.76	.74	.46	.44	.66
Loneliness	35. 00 (1.62)	34.03 (1.54)	33.76 (1.65)	36.18 (1.59)	32.36 (1.48)	30.75 (1.65)	1.47	.23	1.34	.18	1.68	.10

*Note*. Analyzed with Linear Mixed Method. Parent report *N* = 198. Teacher report *N* = 195. Child report *N* = 121.

### Effect size

By convention, an effect size (Cohen’s *d*) of .20 is regarded as a small effect, .50 is considered a moderate effect, and .80 is considered a large effect [45]. The ECBI intensity was the only outcome measure that showed a statistically significant difference between the ISST and the comparison group; it had an effect size on .31, which can be viewed as a small to moderate effect.

## Discussion

The present study was a randomized effectiveness trial of individually delivered child social skills training in a sample of 198 children who had emerging or existing conduct problems. Generally, the findings showed a decrease in conduct problems and internalizing problems, and an increase in social competence across the ISST and the comparison group from intake to six months after the termination of the intervention. The ITT results from this trial showed that one of ten outcomes was significantly in favor of ISST immediately after the intervention, while none were significant six months later (at follow-up). The immediate significant positive effect of ISST emerged in the parent ratings of child conduct problems (ECBI intensity).

It is interesting that the only effect in this study emerged in parent reports. Although this effect only was found on one of three measures of conduct problems, this could suggest that children’s negative behaviors may decrease at home as a result of ISST. However, the most striking finding from this trial was the lack of significant effects. This finding corresponds with the results of the meta-analyses of single-case studies of individually delivered social skills training [23,24]. The present study tested ISST in an effectiveness trial with the use of a relatively large sample, which strengthens the external validity of the findings.

The lack of positive effects may have several causes. First, and in accordance the principles of TIBIR, it may be the case that individually delivered social skills training is not sufficient to reduce conduct problems in children. Theoretically, as suggested by the SIL-model [10], child conduct problems are caused by coercive parent-child interactions. It may be an inherent weakness in ISST that it does not address parenting practices. Even if targeting children directly may have an impact [22], our data suggest that neither the parents (with one exception), the teachers, nor the children themselves perceived any positive effects of ISST. Perhaps parents and teachers were unaware of the changes in the children’s behavior and therefore did not support their newly acquired social skills. In that case, no generalization of the skills learned during ISST would be expected. If these children were still involved in coercive interactions at home and at school, the addition of parent and teacher training would most likely be needed for ISST to have an impact on their conduct problems. At least, the inclusion of practices with parents, teachers or peers, preferably in the natural context, may have resulted in generalization effects. Also, data on homework completion could possibly have explained the lack of generalization. Previous research has shown that child social skills training combined with a parent or teacher intervention has more beneficial effects on child conduct problems compared to providing a parent or teacher training intervention alone [7,17]. Therefore, the findings from this study suggest that ISST should not be used as the sole intervention. Rather, it should be provided in addition to parent or teacher training. As the present study examined the unique impact of ISST, and not in combination with parent or teacher training, it is premature to conclude whether this intervention may enhance the effects to parent or teacher training. Therefore, more research is needed to evaluate whether ISST may produce additive effects.

A second cause for the lack of positive effects may be that ISST is missing vital components for bringing about change in children with conduct problems. In other words, the lack of effects may be due to the characteristics of the intervention, and not because it was delivered alone. The dosage of the intervention may have influenced the outcomes. On average, ISST lasted for 8.59 one-hour sessions. This dosage may not have been sufficient to produce a considerable impact on child conduct problems. Moreover, it may be the case that children would have benefited from having time to practice their newly acquired skills in the kindergarten and school contexts. Practicing the components in real-life settings has been found, in parent and teacher training interventions, to positively influence outcomes [46,47]. The same may apply to ISST. Webster-Stratton [48] has also argued for including behavioral corrections and specific consequences for negative behavior in the process of teaching social skills. In addition to promoting social skills and ignoring unwanted behavior, the trainers may need stop-mechanisms to reduce aggressive behavior.

A third possibility is the occurrence of measurement problems that may have occurred due to a lack of fit between the social skills assessment instruments and the intervention components. Both SSRS and HCSBS/SSBS are wide-ranging measures of social skills that cover a broader range of skills than those addressed in ISST. Given that the training programs covered only some of the skills measured by the social skills ratings scales, the program’s effectiveness may have been underestimated. A mismatch between the program content and the internalizing problems measured in the CBCL and TRF may also explain the lack of positive outcomes on child problem behavior in general and externalizing problem behavior in particular. As seen in Table 1, there was a slight increase in social skills as assessed by parents and teachers from the program start to the follow-up, but these were matched by a similar trend in the comparison group. However, a closer examination of the correspondence between the skills addressed in the program and in the assessments should be closely scrutinized in future studies.

### Limitations and future directions

The present study had several advantages, including a low attrition rate and a multi-informant design (parents, teachers and children). However, observational measures should have been included to reduce the bias associated with the methods and sources of information used in this study. For example, practitioner adherence to ISST was based on self-reports. Bias related to the use of this measure (e.g., social desirability) could have been reduced with the use of observational data. Moreover, observational data may have shed light on micro-social peer interactions taking place in, for instance, playgrounds. Because parents and teachers do not always observe children when interacting with peers in the preschool and school environment outside the classrooms, the data used in the present study does not assess such interactions. Thus, more research is needed to assess potential changes in peer social interactions following ISST. The current study included a relatively large sample. However, as suggested by a meta-analysis [22], it may not have provided adequate power to detect intervention effect of ISST. The findings should therefore be interpreted with caution, as the sample size increases the likelihood for type 2 errors (i.e., failure to reject the null hypothesis). There was a significant differential attrition rate for the participants in the two groups. More families in the ISST group participated throughout the study, and this factor may have biased the outcomes in this study. Also, as the effectiveness of ISST may have been influenced by the efficacy of the ISST interventionists, the possible clustering of outcome by trainer should have been investigated.

The intervention tracking process for the comparison group was not optimal and should have been more detailed. Although the teachers in the comparison group were free to seek and receive other services, to the best of our knowledge, only 18.6% of the children in the comparison group received any intervention at all. However, the low proportion of children receiving intervention likely reflects the heterogeneity of real world practice and suggests that schools and preschools in many instances do not initiate structured interventions when faced with the challenges of child conduct problems.

## Conclusions

Over the years, competence promotion programs have changed in nature and scope [28]. First-generation efforts were characterized by child-focused definitions of competence and emphasized building singular or core sets of skills [49]. The programs documented that skills could be changed, but the consequences for adjustment turned out to be small in magnitude. Similarly, the present study was not able to demonstrate positive outcomes on child social skills or problem behaviors. Correspondingly, Masten and Coatsworth [28] stated that the most promising programs are complex competence-enhancement approaches in which children are trained in a variety of elaborate skills over long periods of time and links the teaching of skills to the developmental stage of children and to their developmental contexts. The impact of ISST may increase if the intervention is restructured to incorporate the features suggested by Masten and Coatsworth [28].

As noted above, ISST is one of the interventions in TIBIR [9]. The findings from the current trial raise the question of whether ISST effectively reduces child conduct problems. The training protocol does seem to have limited unique effects, which suggests that this intervention should not be offered as the sole intervention for children with conduct problems.
